# Invariance of the PAINAD Scale Between the Black and White Residents Living With Dementia

**DOI:** 10.3389/fpain.2021.757654

**Published:** 2021-12-02

**Authors:** Barbara Resnick, Kimberly Van Haitsma, Ann Kolanowski, Elizabeth Galik, Marie Boltz, Jeanette Ellis, Liza Behrens, Karen Eshraghi, Cynthia L. Renn, Susan G. Dorsey

**Affiliations:** ^1^Pain & Translational Symptom Science, University of Maryland School of Nursing, Baltimore, MD, United States; ^2^Pennsylvania State University, University Park, PA, United States

**Keywords:** pain, dementia, measurement, race, Rasch analysis

## Abstract

The purpose of this study was to test the reliability and validity of the Pain Assessment in Advanced Dementia (PAINAD) and particularly consider whether or not this measure was invariant when used among the Black and White residents. Baseline data from an implementation study testing that included a sample of 553 residents, 30% of who were Black, from 55 nursing were included in this study. The Winsteps statistical program was used to perform the Rasch analysis and evaluate the reliability and validity of the measure based on internal consistency, infit and outfit statistics, mapping, and a differential item functioning (DIF) analysis. The AMOS statistical program was used for confirmatory factor analysis. The findings supported the reliability and validity of the PAINAD when used with these individuals and demonstrated that there was no evidence of invariance between the Black and White residents. All the items fit the model, but there was not a good spread of the items across the pain level of the participants. The majority of the participants (75%) were so low in pain signs or symptoms that they could not be differentiated. Based on the clinical practice and observations, it is recommended that additional items can be added to the measure such as observing the individual for evidence of resisting care, retropulsion when trying to stand, hitting or kicking when turning in bed, hitting or kicking when transferring from bed to chair, hitting or kicking when ambulating, or hitting or kicking when raising arms, less engagement with others, and decreased participation in the activities previously enjoyed.

## Introduction

Approximately half of the individuals living with dementia experience pain (1–4). Unfortunately, pain in this population is difficult to evaluate and often goes unrecognized and untreated. Untreated pain can lower quality of life, negatively impact function, impair sleep, and increase the psychological symptoms associated with dementia including depression, agitation, aggression, and resistiveness to care (5, 6). Challenges to the identification of pain are associated with the difficulty in individuals living with dementia have incommunicating pain verbally (4) along with the timing of the assessment and whether the individual is at rest or engaged in some type of activity (7). To help overcome the verbal challenges associated with reporting pain, observation measures are recommended when evaluating pain in older adults living with dementia (8).

### Observational Pain Assessment Tools

Multiple systematic reviews have been done to address the assessment of pain in older adults with dementia. In a 2016 review (9) of 23 papers, 6 tools were described as self-rated and 18 tools were described as observation measures. The findings from this review indicated that self-report should be used when all possible observations are used for supplementation of self-report. In a 2014 systematic review article (10) of 28 pain assessment tools for the individuals with dementia, it was noted that none had sufficient evidence of reliability and validity and, thus, there was no recommendation for the use of one tool over any of the others. In an earlier review of just 12 observation measures (11), three were recommended for the potential use with older adults living with dementia as these three measures had the strongest evidence of reliability and validity. The recommended measures included the Pain Assessment Checklist for Seniors with Limited Ability to Communicate (PACSLAC), the Pain Assessment in Advanced Dementia (PAINAD), and the Mahoney Pain Scale (MPS) (11). The PAINAD has some advantages over the other tools in that it is simple to utilize and requires minimal, if any, training (12). The PAINAD scale includes the categories and behaviors associated with pain included in other measures and also noted in the literature and based on expert consensus. It includes five items: breathing, negative vocalization, facial expression, body language, and consolability. Responses range from 0 to 2 on each item with higher scores indicative of more pain/discomfort based on the descriptions of the behavior one expects to see at that level of pain (for example, 0 would be body language relaxed and 2 would be body rigid, fist clenched, knees pulled up, pulling or pushing away, or striking out). Total scores range from 0 (no pain) to 10 (severe pain). Multiple studies have supported the reliability and validity of this measure (13–15).

Conversely, the PACSLAC is more comprehensive and includes 60 items incorporated within five subscales: facial expression, activity and body movement, social personality, and mood, and other behaviors (e.g., changes in eating and sleeping). Each item is scored as present or absent and then a total score is summed with higher scores indicative of more severe pain. This measure has been shortened and tested as a 31-item measure referred to as the PACSLAC-II (16). Although there is support for the reliability and validity of the PACSLAC and the PACSLAC-II (16–18), the measure requires more training and takes more time to complete than the PAINAD.

The third observation measure recommended was the MPS (19). The goal of this measure was to identify evidence of pain and pain severity and differentiate pain from agitation. A total of eight items are included within two subscales. Responses range from 0 which refers to minimal pain to 3 which refers to severe pain. The first subscale focuses on pain-related behaviors such as facial expression and the second subscale includes items that differentiate pain from agitation. Scores are summed to indicate little pain, mild, moderate, or severe pain. There is some evidence of the reliability and validity of this measure (19), although less psychometric testing has been done with the other measures. Therefore, this measure further assumes that there will be some pain, which may not be the case with all the residents.

### Racial Disparities and Pain

Although findings are not always consistent, it has been reported that the Black residents in nursing homes experienced more pain and were less likely to be treated for pain compared to the White residents (3, 20, 21). Numerous racial differences in pain perception, response to and coping with pain, and pain treatment have been described. Independent of age, sex, socioeconomic status, education, and medical comorbidities, Black older adults have reported more pain-related disability compared to White older adults (22–25). Further, Black older individuals have lower self-efficacy related to the management of pain and more depression due to the pain (23). Although studied mostly with the younger adults, enhanced physiological pain sensitivity in the minority groups, particularly between the Black and White adults, has been demonstrated by using the quantitative sensory testing methods looking at thermal pain, cold pressor pain, ischemic pain, electrical pain, and conditioned pain modulation (26, 27). The underlying mechanisms for these differences include (1) psychophysiological factors such as reduced nociceptive flexion reflex thresholds (27); (2) genetic differences including such things as differences in stress-induced pain regulatory mechanisms involving blood pressure, norepinephrine, and cortisol all of which function more effectively among the White vs. Black adults; and (3) sociocultural issues or social determinants, which impact the meaning that the individual ascribes to the pain and how they respond to the sensation of pain with the Black individuals having a stronger link between the emotions and pain than the White adults. Social determinants that influence pain include economic stability, environment, education, social context and community, and the healthcare system (28–32). Consistently, there are disparities between the social determinants between the Black vs. White older individuals such as lower education levels increase the risk of experiencing long-term chronic pain after orthopedic events and oral care (30–32).

### Racial Differences in Pain Signs and Symptoms

Black adults are more likely to cope with pain by obtaining social support, focusing on prayer, catastrophizing, and avoiding the pain or by using distraction to cope with the pain vs. using active approaches such as exercise or seeking out medical management (33–36). Black adults with chronic pain tend to have more depressive symptoms and symptoms similar to posttraumatic stress disorders, more sleep disturbance, and more complaints of comorbidities compared to White adults with chronic pain (37).

Based on the differences in pain presentation and interpretation and coping among the Black vs. White older adults, it is possible that the presentation and observations of pain symptoms between the Black vs. White residents living with dementia may be different. Although the observation measures used previously have evidence of reliability and validity, they have not been tested for invariance across the different racial or ethnic groups. The purpose of this study was to test the reliability and validity and invariance of the PAINAD when used with Black and White residents with moderate to severe dementia. The PAINAD was used in this study because of the consistent and strong psychometric properties when used with older adults with dementia and the ease at which it is completed. Gaining a better understanding of the use of this measure across the racial groups will help to assure that pain can be accurately identified among the Black and White residents with moderate to severe dementia in these settings.

## Methods

### Design

This was a descriptive study using baseline data from the Evidence Integration Triangle for Behavioral and Psychological Symptoms of Dementia (EIT-4-BPSD) implementation study. Data were obtained between 2017 and 2020. The study was approved by a University-based Institutional Review Board and has been previously described and major findings are reported (38).

### Sample

A total of 55 nursing homes from two states participated in this study. These facilities had to: (1) agree to actively partner with the research team on an initiative to change practice; (2) have at least 100 beds or at least 50 beds if the facility was a dedicated dementia care unit; (3) provide a staff member to be an internal champion and work with the research team in the implementation of an approach to care that increased the use of person-centered approaches for the management of behavioral and psychological symptoms associated with dementia; and (4) be able to access email and websites *via* a phone, tablet, or computer. To participate in this study, the residents had to: (1) live in a participating nursing home; (2) be 55 years of age or older; (3) have cognitive impairment based on a score of 0–12 on the Brief Interview of Mental Status (BIMS) (39); (4) not be enrolled in hospice or admitted for short-stay rehabilitation. The Evaluation to Sign Consent (ESC) was given to all the potential participants and if this was not passed by answering items correctly (40), the individual had to assent to participate and the legally authorized representative (LAR) was invited to complete the consent process. In this study, 1,100 residents were approached; 43 (4%) LARs of the 1,100 approached residents were non-communicative, did not understand English, died, or were transferred before they could consent; 156 (14%) LARs refused to assent or consent to participate; 221 (20%) LARs were unavailable; and 90 (8%) LARs refused to consent. A total of 572 residents consented and 19 residents were not eligible, as they had a BIMS score >12; 11 residents were ineligible, as they were on hospice; and 7 residents were ineligible, as they were younger than 55 years leaving 553 residents enrolled into this study. Full baseline data were obtained on 536 participants, as 17 residents died or were transferred out of the setting prior to the data collection.

### Procedure and Measures

Data collection were completed by the trained research evaluators based on chart extraction, direct observation of the resident, and input from the clinical staff working with the resident on the day of testing. Descriptive information included age, race, gender, cognitive status, and comorbidities. The number of comorbidities was obtained based on a sum of the 13 comorbid conditions described within the Cumulative Illness Rating Scale (41). Cognitive status was based on the BIMS (39) with scores of 8–12 indicative of moderate cognitive impairment and scores 0–7 indicative of severe cognitive impairment.

The pain was evaluated by using the PAINAD described above. Evaluators were provided with a list of observable behaviors for each of the five items and told to observe the resident during a period of activity such as bathing, dressing, walking to the bathroom, or dining room. A cutoff score of 2 or more was considered as indicative of pain (8).

### Data Analysis

Descriptive statistics were done by using the Statistical Package for the Social Sciences (SPSS) version 24.0 (Armonk, New York, NY, United States), the Winsteps statistical program was used to perform the Rasch analysis, and the AMOS statistical program for confirmatory factor analysis. Testing for unidimensionality was done by evaluating a principal components factor plot and providing evidence that the first factor explained <15% of the residual variance (42). Unidimensionality was also considered based on evidence of good model fit in confirmatory factor analysis.

### Reliability Testing

Internal consistency of the PAINAD was evaluated based on item reliability by using the Rasch measurement model (42). The person separation index is provided, which is equivalent to an alpha coefficient (43). Evidence of internal consistency was considered sufficient if the alpha coefficient was 0.7 or greater (43). A differential item functioning (DIF) analysis was done to assure that the items on the measure were reliable across the racial groups. DIF > 1.00 logit is considered meaningful and is determined to be significant at a level of *p* < 0.05.

### Validity Testing

Construct validity was done by using a Rasch measurement model to demonstrate that the items fit the data. Item fit is based on the infit and outfit statistics with a range between 0.4 and 1.6 that are considered as appropriate (44). An infit or outfit value that is < 0.4 may mean that the item is redundant and not adding anything to the explanation of the concept and values >1.6 may be measuring another concept aside from pain (42). Item mapping was also used to consider the validity of the PAINAD. Mapping provides information about the spread of the items across the concept of pain in older adults with dementia and provides assurance that the items are not too difficult for the participants to endorse or demonstrate or too easy to endorse or demonstrate when in pain. Good validity is established if there is a close fit between the mean item measure with the mean person measure on the map (45).

To consider the construct validity and invariance of the measure across the Black vs. White participants, the sample was divided by race, and a confirmatory factor analysis was performed. The sample covariance matrix was used as input and a maximum likelihood solution was sought. Model fit was based on the chi-squared statistic divided by the degrees of freedom, the normed fit index (NFI), and the Root Mean Square Error of Approximation (RMSEA) (46). The chi-squared statistic divided by the degrees of freedom of 5 or less, the NFI close to 1.0, and the RMSEA of <0.10 provide evidence of good model fit (46, 47). Evidence of path significance was established, if the critical ratio (CR), which is the parameter estimate divided by an estimate of the SD, was > 2 in absolute value (46). *p* < 0.05 level of significance was used for all the analyses. To consider if there was invariance between the fit of the items to the data when tested with the Black vs. White participants, the models were compared for the significant changes in the chi-squared statistic divided by the degrees of freedom and improvements in the NFI and the RMSEA (46).

## Results

Among the 55 facilities, 27 (49%) had no Black residents in the study and the remaining 28 facilities (51%) had anywhere from 7 to 100% of the participants being Black residents. As shown in Table 1, the mean age of the participants was 83.88 years (SD = 10.45), the majority were females (72%) and White (76%), and overall they had a mean of 7.10 (SD = 2.16) comorbid conditions. The Black participants were younger than the White participants with a mean age of 78 (SD = 11) vs. 86 years (SD = 9), had fewer comorbidities [White participants 7.26 (SD = 2.18) and Black participants 6.60 (SD = 2.02)] and were more likely to be men (43 Black male participants vs. 23% White male participants). There was no difference in the BIMS score between the Black and White participants with an overall mean of 4.29 (SD = 3.45).

**Table 1:** Description of sample.

**Variable**	**Mean (SD)**	***N*** **(%)**
Age	83.88 (10.45)	
Cognition (brief inventory of mental status)	4.29 (SD = 3.45)	
Comorbidities	7.10 (2.16)	
PAINAD	0.68 (1.50)	
Evidence of pain		86 (16%)
No pain		450 (84%)
Gender		
Male		155 (28%)
Female		398 (72%)
Race		
Black		135 (24%)
White		419 (76%)

### Unidimensionality and Reliability Results

The results of the principal components factor plot showed that the first factor explained 30% of the residual variance suggesting the possible multiple dimensions. The confirmatory factor analysis, however, showed that all the items loaded significantly onto the concept of pain as shown in Table 2, although the factor loading for breathing was 0.34, which is much less than the preferred 0.50–0.70 (46, 48). There was evidence of internal consistency based on an equivalent alpha coefficient of 0.95. The DIF analysis is shown in Table 3. There was no evidence of DIF on any of the items between the White and Black participants. DIF size was <1 logit across all the items.

**Table 2:** INFIT and OUTFIT statistics for the PAINAD scale, mapping order, and factor loadings for the full measurement model.

**Item**	**Description of the item**	**INPUT** MNSQ (ZSTD)*	**OUTPUT** MNSQ (ZSTD)*	Mapping order**	**Factor loadings**		
					**Full** **sample**	**Black** **sample**	**White** **sample**
Breathing	0-normal 1-occasional labored breathing and short period of hyperventilation 2-noisy labored breathing or long period of hyperventilation.	1.26 (1.35)	1.53 (1.61)	5	0.34	0.34	0.34
Negative vocalizations	0-none 1-occaisional moan or groan or low level of speech with a negative or disapproving quality 2-repeated troubled calling out or loud moaning, groaning, or crying	1.09 (0.85)	1.06 (0.53)	4	0.75	0.75	0.75
Facial expression	0-smiling or inexpressive 1-sad, frightened, frown 2-facial grimacing	0.97 (−0.18)	1.02 (0.16)	1	0.69	0.69	0.69
Body language	0-relaxed 1-tense or distressed pacing or fidgeting 2-rigid, fists clenched or knees pulled up, pushing or pulling away or striking out at others	0.91 (−0.17)	0.87 (−0.93)	2	0.68	0.68	0.68
Consolability	0-no need to console 1-distracted or reassured by voice or touch 2-unable to console, distract or reassure	0.79 (−1.85)	0.83 (−1.34)	3	0.80	0.80	0.80

* *Mean Square (standardized estimate)*.

** *Mapping order from 1 (easiest to endorse or most commonly noted) to 12 (most difficult to endorse or least commonly noted)*.

**Table 3:** DIF analysis for evidence of pain across racial groups.

**Person** **classification**	**Scale item**	**DIF score**	**DIF measure**	**DIF size**	**DIF SE**	**DIF t**	***P***
**Race**							
White	Breathing	0.01	1.55	−0.09	0.26	−0.35	0.73
Black	Breathing	−0.04	2.04	0.40	0.57	0.70	0.49
White	Negative vocalization	0.06	−0.30	−0.18	0.18	−0.97	0.34
Black	Negative vocalization	−0.17	0.57	0.69	0.39	1.79	0.08
White	Facial expression	−0.02	−0.81	0.05	0.18	0.31	0.76
Black	Facial expression	0.06	−1.03	−0.17	0.31	−0.55	0.58
White	Body language	−0.02	−0.36	0.07	0.18	0.37	0.71
Black	Body language	0.06	−0.64	−0.21	0.32	−0.65	0.52
White	Consolability	−0.03	−0.13	0.10	0.19	0.53	0.60
Black	Consolability	0.09	−0.54	−0.31	0.32	−0.95	0.35

### Validity Results

The items that all fit the data with infit and outfit statistics ranging from 0.79 to 1.53 (Table 2). Mapping results are shown in Table 2 and Supplementary Figure 1. The easiest item to endorse or not related to pain was facial expressions, then body language, then consolability, then negative vocalizations, and changes in breathing were the hardest or most infrequent behavior noted with regard to pain. The majority of the participants (*n* = 416 or 75%) were so low in pain signs or symptoms that they could not be differentiated. There were no individuals so high in pain that they could not be differentiated. The match between the pain of the person and the difficulty of the items was fair with the means being 1.3 logits apart.

The fit of the model to the data was fair with a χ^2^/df ratio of 7.22 overall, the NFI of 0.96, and the RMSEA of 0.11. There was no difference in fit between the Black vs. White participants, as there were no differences in any of the indices between the races.

## Discussion

There was some evidence to support the uni-dimensionality of the PAINAD and analyses were performed by assuming unidimensionality. The unexplained residuals were high at 30% and there was one item, the item focused on breathing, which had a factor loading that was < 0.50–0.70 in the full sample as well as when used with the Black and White participants only. The assumption of unidimensionality was based on the fact that there were only five items in the measure and subscales with less than at least three items would not have been appropriate (46).

The reliability of the measure was well-supported based on evidence of internal consistency with an alpha coefficient of >0.70 which is the cut-off for an acceptable level of internal consistency (43). Likewise, there was support for the reliability of the measure as there was no evidence of DIF across any of the items for the Black vs. White participants. The lack of a difference in the use of the PAINAD across races was also supported by invariance of model fit when tested with the White only vs. Black only participants. Model fit and factor loadings were exactly the same across both groups. The PAINAD, however, includes only five signs of pain and maybe missing the presentation of other signs of pain in the Black vs. White older individuals such as the impact of pain on function and the ability to overcome or manage the pain (22–25).

Multiple prior studies have also provided support for the reliability and validity of the PAINAD when used with the multiple ethnicities (49, 50) and across many clinical conditions (11, 13, 14, 51, 52). These studies did not, however, compare use across the Black vs. White older adults and did not use a Rasch analysis approach.

The low level of pain noted in the participants may have been related to the timing of the assessments. Medication management may have contributed to the low levels of pain, but only 28 participants (5%) were receiving opioids for the treatment of pain (53). We do not know if behavioral interventions to manage pain were used and this also may have influenced levels of pain. The evaluators were encouraged to complete the PAINAD during times of activity, as this is likely when these individuals would demonstrate the commonly noted signs of pain. It is possible, however, that the participant was observed walking, but the pain only occurred for that individual when he or she was bathing or dressing. Completion of the measure during a variety of activities might be helpful, as was done in a study by Bargellini et al. (7). According to the study by Bargellini et al. (7), the PAINAD was completed at rest, repositioning in bed, transferring from bed to standing, from bed to chair, or during a treatment that was potentially painful (e.g., wound care). Alternatively, pain could be more comprehensively tested by inducing pain by using quantitative sensory testing methods looking at thermal pain, cold pressor pain, ischemic pain, electrical pain, and conditioned pain modulation (26, 27) and adding these responses to the PAINAD. Completion of quantitative sensory testing in individuals with moderate to severe dementia, however, may be challenging for ethical reasons and the procedures need to be adjusted due to the ability of the participants to follow the instructions associated with testing.

There was also some support for the validity of the measure based on the good infit and outfit statistics and factor loadings in the confirmatory factor analysis were all acceptable with the exception of the item for breathing, which was significant but just 0.34. Differences in breathing in older adults may be due to multiple physiological factors such as congestive heart failure, chronic obstructive pulmonary disease, or deconditioning. As it has been previously noted (54), this item may not be the best indication of pain. Alternatively, it might be better for the evaluators to observe the participant for an increased respiratory rate with activity, a decrease in pulse oximetry, or shallow respirations indicative of guarding against pain or coping with the pain.

Although all the included items fit the model, there was not a good spread of the items across the pain level of the participants. The majority of the participants (75%) were so low in pain signs or symptoms that they could not be differentiated. It is possible that the majority of the study participants did not have pain, although this is not consistent with prior studies suggesting that 33–80% of nursing home residents with moderate to severe dementia were noted to have pain based on observation (7, 8, 55). Additional items for the PAINAD may be needed to better differentiate individuals who have pain, so as not to miss pain presentation in those who cannot communicate this symptom. Additional items recommended based on the clinical experience and observations include such things as evidence of resisting care, retropulsion when trying to stand, hitting or kicking when turning in bed, hitting or kicking when transferring from bed to chair, ambulating or when raising arms, less engagement with others, and decreased participation in activities previously enjoyed. Further, it may be helpful to delineate the items under the current categories included in the PAINAD such as adding things such as frowning or looking tense for facial expression or shouting or groaning under vocalization as done in several recently developed observational pain measures (2, 56).

## Conclusion

This study supported the reliability and validity of the PAINAD measure when used with older adults with moderate to severe cognitive impairment and showed that in the current form, there was no invariance in the use of the measure across racial groups. The findings, however, suggest that it would be useful to add the additional items to the measure to more comprehensively address observational pain in the older adults with moderate to severe dementia who are generally unable to express verbally that they are experiencing pain. Of particular importance, they need to evaluate pain during the different types of activity that may cause pain in this population such as turning in bed, transferring, or ranging upper extremities.

### Strengths and Limitations of the Study

Some limitations in this study need to be acknowledged. Although the sample was large and included residents from 55 settings, it was done in only two states and participants had to assent or consent to participate and, thus, we may not have recruited those that were agitated due to pain to the point that they would not assent. Evaluators were instructed to observe the resident during times of activity, but this was not standardized or done across the different types of activities for all the participants. Further pain evaluations were done only during a brief single time point and we did not gather data on the use of non-pharmacologic interventions that may have been used or adjust for use of treatment for pain either pharmacologic or non-pharmacologic. Overall, the current sample had very low levels of pain, which was not consistent with what is generally reported in nursing home residents. Although the percentage of Black residents included in this study was higher than the percentage of Black residents in the United States, this study may have benefitted from a larger percentage of the Black participants. Comorbidities were considered based on the total number of conditions noted. Future research might benefit from including only individuals with diagnoses noted to be associated with chronic conditions that commonly cause pain such as osteoarthritis. Despite these limitations, the findings support that the PAINAD is a useful measure to evaluate pain in a population in which verbal reporting is challenging. Some revisions to the measure are recommended to strengthen the identification of pain and value of this measure for future use and to provide better insurance of invariance across racial groups.

## Data Availability Statement

The raw data supporting the conclusions of this article will be made available by the authors, without undue reservation.

## Ethics Statement

The studies involving human participants were reviewed and approved by University of Maryland School of Medicine. The patients/participants provided their written informed consent to participate in this study.

## Author Contributions

All authors have contributed to the data collection, design, analysis, and interpretation and write up of this manuscript.

## Funding

This study was funded by the National Institute of Nursing Research, United States (R01 NR015982).

## Conflict of Interest

The authors declare that the research was conducted in the absence of any commercial or financial relationships that could be construed as a potential conflict of interest.

## Publisher's Note

All claims expressed in this article are solely those of the authors and do not necessarily represent those of their affiliated organizations, or those of the publisher, the editors and the reviewers. Any product that may be evaluated in this article, or claim that may be made by its manufacturer, is not guaranteed or endorsed by the publisher.

## Supplementary Material

Supplementary Figure 1 Item mapping.
