# Supplementary figures and images for: Invariance of the PAINAD Scale Between the Black and White Residents Living With Dementia
Source: Front Pain Res (Lausanne). 2021 Dec 2;2:757654. doi: 10.3389/fpain.2021.757654 (PMC8915686; doi:10.3389/fpain.2021.757654)

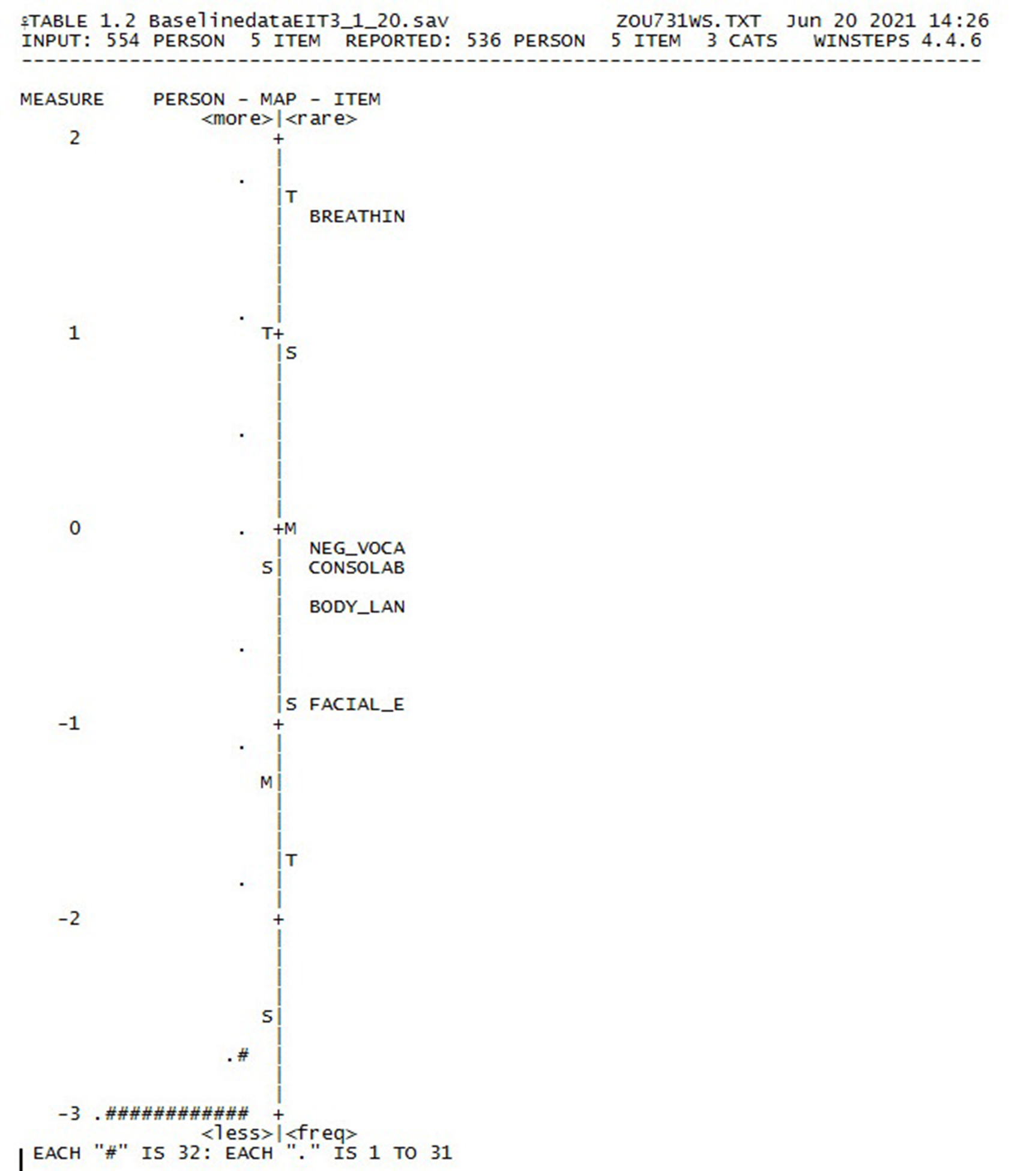

Supplement: Supplementary Figure 1 — Item mapping. [file Image_1.jpg]
